# Laparoscopic cholecystectomy for a cholelithiasis patient with an aberrant biliary duct of B5: a case report

**DOI:** 10.1186/s40792-020-00981-z

**Published:** 2020-09-30

**Authors:** Takuto Suzuki, Yoh Asahi, Akifumi Sawada, Kohei Umemoto, Masaya Kina, Masahiro Shinohara, Kazunori Yokoyama, Hiroyuki Masuko

**Affiliations:** 1 Department of Gastroenterological Surgery, Nikko Memorial Hospital, 1-5-13, Shintomi-cho, Muroran, Hokkaido 051-8501 Japan; 2 Department of Radiology, Nikko Memorial Hospital, 1-5-13, Shintomi-cho, Muroran, Hokkaido 051-8501 Japan; 3 Department of Gastroenterology, Nikko Memorial Hospital, 1-5-13, Shintomi-cho, Muroran, Hokkaido 051-8501 Japan

**Keywords:** Aberrant biliary duct, Bile duct injury, Biliary tree, Cholelithiasis, Endoscopic retrograde cholangiopancreatography, Gallbladder, Intraoperative complications, Laparoscopic cholecystectomy, Segment 5

## Abstract

**Background:**

An aberrant biliary duct of segment 5 (B5) is a rare anomaly of the biliary tract. All anatomical anomalies of the biliary tract are risk factors for bile duct injury during surgery. We report a case of cholelithiasis with an aberrant B5 that was detected during a detailed preoperative imaging examination and treated with laparoscopic cholecystectomy.

**Case presentation:**

A 69-year-old woman was admitted to the emergency room of our hospital with abdominal pain. She was diagnosed with cholelithiasis, and an aberrant B5 branching off the hepatic duct was suggested during preoperative imaging. Laparoscopic cholecystectomy was performed at our surgical department. There were no intra- or postoperative complications, and the patient was discharged on the fourth day after surgery.

**Conclusions:**

Laparoscopic cholecystectomy can be safely performed without intra- or postoperative complications in patients with cholelithiasis and an aberrant B5 if it is accurately diagnosed preoperatively.

## Background

Laparoscopic cholecystectomy (LC) is the surgical gold standard for the treatment of symptomatic gallbladder stones. Bile duct injury during LC is a severe surgical complication that surgeons aim to prevent, because it sometimes requires conversion to laparotomy or bile duct reconstruction. Moreover, bile duct injury can be fatal [1]. The incidence of bile duct injury during LC is 0.11–0.74% [2–4], and insufficient surgical training, excessive tissue inflammation, intra-abdominal adhesions, concurrent biliary disease, and rare anatomical anomalies of the biliary tract are reported to be risk factors [4–7]. Although the term is not strictly defined, “aberrant hepatic duct” is often used to describe an anatomical anomaly of the biliary tract that drains a hepatic segment before merging into the common hepatic duct, common bile duct, or cystic duct [8]. An aberrant biliary tract of B5 is extremely rare, with only three reported cases so far [9–11]. We describe a case of cholelithiasis with an aberrant biliary duct of segment 5, which was preoperatively diagnosed and successfully treated by LC.

## Case presentation

**Fig. 1:**
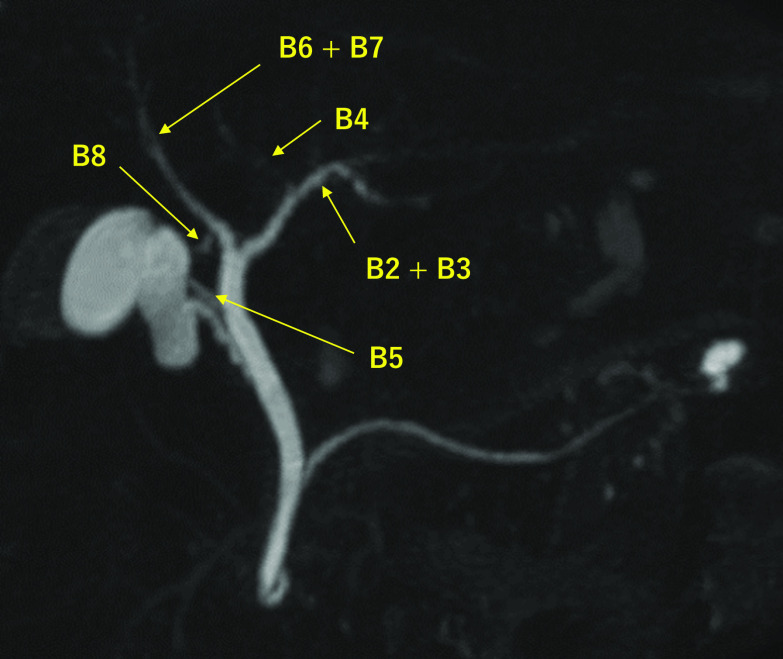
A 69-year-old woman presenting with abdominal pain and gallbladder stone in ultrasound examination. Magnetic resonance cholangiopancreatography showing an aberrant biliary duct of liver segment 5 branching directly off the common hepatic duct

**Fig. 2:**
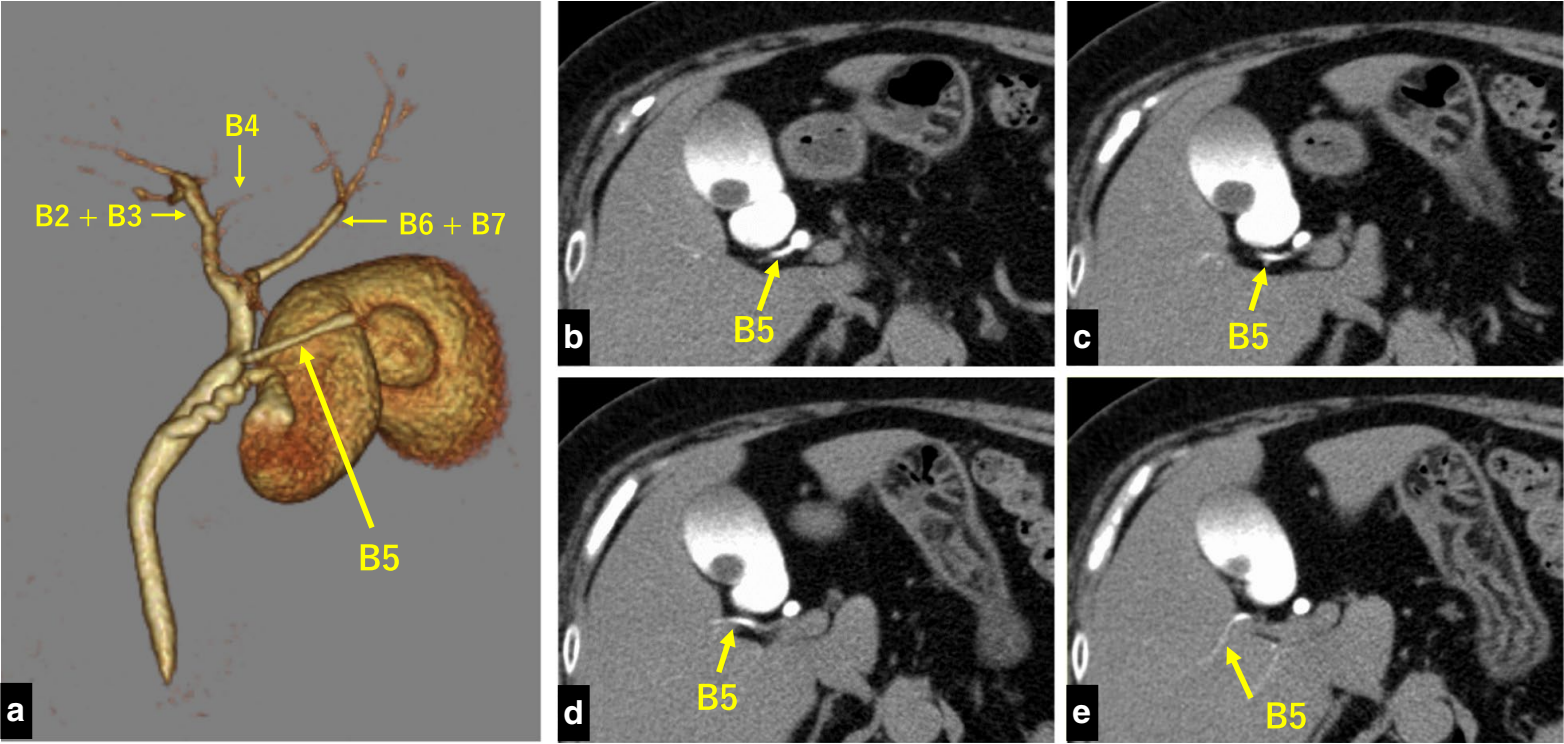
A 69-year-old woman with cholelithiasis and aberrant biliary duct draining segment 5. Drip-infusion cholangiography-computed tomography accurately demonstrating the aberrant biliary duct of segment 5 branching directly off the common hepatic duct near the confluent section of the cystic duct and the common hepatic duct

A 69-year-old woman was admitted to the emergency room of our hospital with abdominal pain. She had developed epigastric pain after breakfast that extended across the entire abdomen with time. The patient had no other abdominal symptoms, such as nausea, diarrhea, or constipation. The patient had had appendectomy for appendicitis in her teens but had no other medical history. Her height was 159 cm and body weight was 83 kg. The patient had no high-grade fever, and other vital signs were in the normal range. The abdomen was soft and flat, although localized tenderness without rebound was noted in the epigastrium and right upper abdomen. Blood examination was normal, and no abnormal intestinal gas detected on the abdominal X-ray images. Abdominal ultrasonography depicted a 13-mm gallbladder stone with an acoustic shadow in the body of the gallbladder. Enhanced computed tomography (CT) was performed, but there was no thickening or enhancement of the gallbladder wall, and the stone was not visualized. Magnetic resonance cholangiopancreatography (MRCP) showed a signal defect in the gallbladder produced by the stone but no such defect in the common biliary duct. There was no dilation of the common biliary duct, but an aberrant biliary duct of liver segment 5 (B5) was detected, which drained directly into the common hepatic duct near the confluence of the cystic duct and the common hepatic duct (Fig. 1). No other B5 was visible on the image. Drip-infusion cholecystocholangiography CT (DIC-CT) confirmed the findings of the MRCP (Fig. 2). On review of the enhanced CT, no blood vessels around the aberrant B5 could be identified. Esophagogastroduodenoscopy revealed nothing to explain the abdominal symptoms of the patient. She was diagnosed with cholelithiasis and an aberrant B5 draining from the hepatic duct and referred to our department for surgical treatment.

**Fig. 3:**
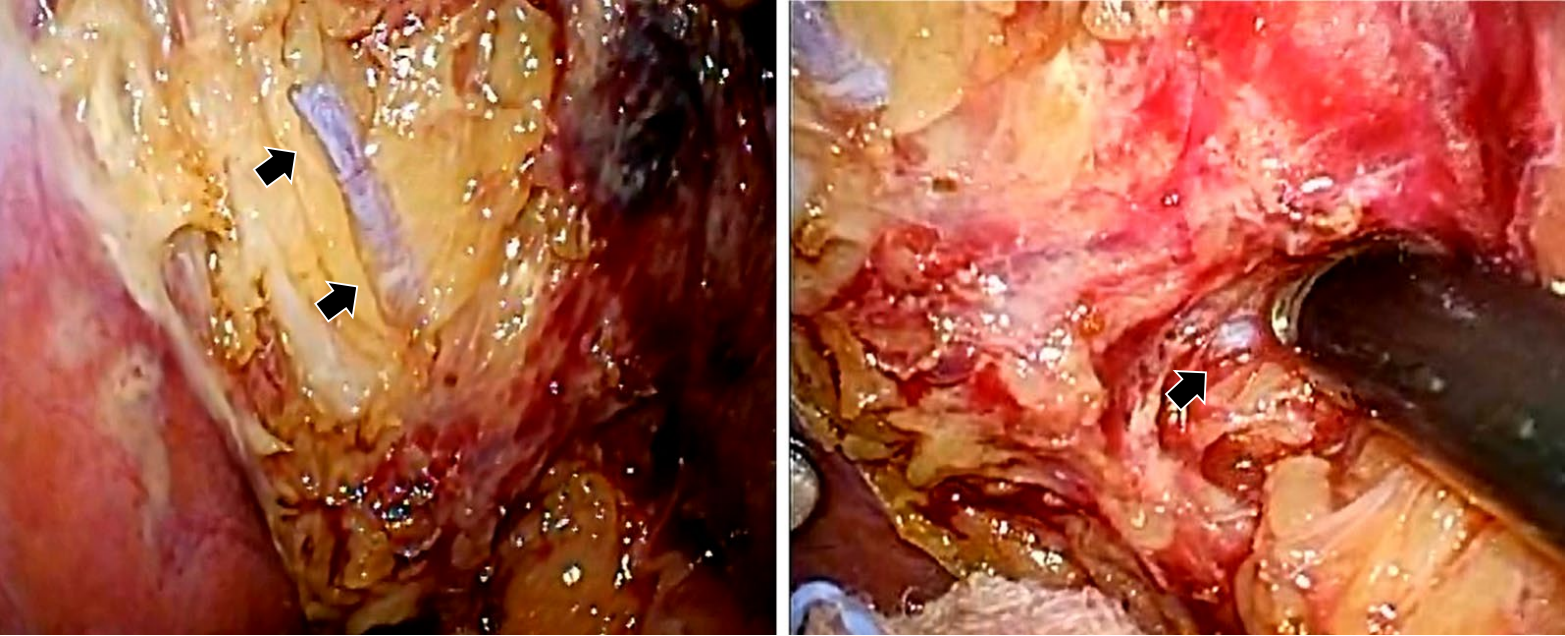
Intraoperative findings in a 69-year-old woman with cholelithiasis and aberrant biliary duct. Static image extracted from the intraoperative video showing the aberrant biliary duct of segment 5 (black arrow) after separation from the gallbladder

Conventional laparoscopic cholecystectomy with four trocars was performed. The HARMONIC ACE^®^+ ultrasonic scalpel (Ethicon Endo-Surgery, Inc., Cincinnati, OH, US) was used during the procedure. Initially, the aberrant B5 could not be identified. The cystic artery was divided with the HARMONIC ACE^®^+, and the cystic duct was severed after double clipping. The serous membrane around the gallbladder was dissected with the HARMONIC ACE^®^+ and separated bluntly from the gallbladder bed. All procedures were performed as close as possible to the gallbladder to avoid damaging the aberrant B5. Consecutively, a tubular structure, which was assumed to be the aberrant B5, was detected in the gallbladder bed (Fig. 3). No signs of injury to the aberrant B5 were visible upon completion of the cholecystectomy, and a subhepatic drain was placed to monitor the abdominal conditions. The operative time was 1 h and 23 min, and blood loss was minimal. No complications occurred after surgery. The subhepatic drain was removed, and oral intake was started on the first postoperative day. The patient was discharged from the hospital on the fourth day.

## Conclusions

An aberrant B5 is a rare abnormality of the bile duct system. Our report describes a case of cholelithiasis in which such an aberrant B5 was identified during the preoperative diagnostic imaging examinations and treated successfully with LC.

Various anatomical abnormalities of the biliary tract have been reported, such as the dorsocaudal branch draining into the left hepatic branch, trifurcation, bile duct duplication, additional accessory bile ducts that intercommunicate with other biliary segments and drain the same areas of the liver, and aberrant hepatic ducts that do not communicate with other biliary segments, but present the only bile ducts draining a particular segment of the liver [8, 12].

The incidence of aberrant hepatic ducts is 4.6% [13]. Biliary duct variations are observed during intraoperative cholangiography in 37–42% [13, 14]. Abnormalities of the right posterior hepatic duct are commonly reported [11], with a right posterior hepatic duct draining into the left hepatic duct being the most common abnormality of the bile duct system observed in 11–13% [13, 14]. Huang et al. [15] developed a classification of biliary tract variations based on the variations of the right posterior hepatic duct, which is commonly used in practice. On the other hand, abnormalities of B5 are rare, with only three cases previously reported. None of these cases underwent laparoscopic cholecystectomy. Akamatsu et al. [9] reported a case of a B5 opening directly into the right posterior hepatic duct, Hyodo et al. [10] reported a B5 opening directly into the common hepatic duct, and Kataoka et al. [11] reported a B5 opening directly into the cystic duct. There are no other reports describing abnormalities of B5, making the present case markedly rare.

The known severe surgical complications of LC include bile duct injury, injury of the abdominal organs, and severe bleeding. Bile duct injury during LC is observed at an incidence rate of 0.11–0.74% [2–4]. Patients with intraoperative bile duct injury can present with unexpected fever, abdominal pain, abdominal distension, nausea, and malaise after the procedure. Bile leakage can cause abscess formation and peritonitis. Moreover, bile duct injury can be fatal if it leads to sepsis or liver failure [1, 16]. The risk factors for bile duct injuries are insufficient training of the surgeon, extensive inflammation or adhesions around the bile duct, complications of other biliary diseases, and anatomical abnormalities of the bile duct [4–7]. The LC in our patient with an aberrant B5 entailed a high risk of bile duct injury, and it is important to clarify the actual bile duct anatomy prior to surgery in these cases.

Imaging studies to evaluate bile duct abnormalities include endoscopic retrograde cholangiography (ERCP), MRCP, and DIC-CT. While ERCP is an effective imaging study for the evaluation of bile duct anatomy, it is highly invasive and causes post-ERCP pancreatitis in 0.09% of patients [17, 18]. Hence, MRCP and DIC-CT are preferred over ERCP, because they are minimally invasive. MRCP is commonly performed to evaluate bile duct anatomy and disease, especially in the case of dilated intrahepatic ducts [10]. The high resolution of DIC-CT enables detailed evaluation of bile duct variations, including small ducts, such as the intrahepatic ducts and aberrant hepatic ducts. It also enables the evaluation of bile duct patency [10]. Kataoka et al. [11] suggested performing MRCP as a routine before elective LC, and recommended performing DIC-CT or ERCP if further examination is needed. Furthermore, they suggested that DIC-CT should be considered in cases with an insufficient illustration of the bile tract on MRCP and ERCP should be considered in cases of choledocholithiasis. In our patient, MRCP had shown the aberrant B5, and we decided to perform DIC-CT to obtain a more detailed understanding of the anatomy, including the relationship between the aberrant B5 and other biliary ducts.

Preoperative insertion of an endoscopic nasobiliary drainage tube or intraoperative placement of a biliary tube through the cystic stump enables intraoperative cholangiography to avoid bile duct injury [19, 20]. We had been prepared to place a biliary tube intraoperatively in our patient, but could complete the LC safely without intraoperative cholangiography, because we were able to precisely identify the aberrant B5.

Honda et al. [21] suggested that the subserosal layer of the gallbladder wall is divided into an inner and outer layer, and that aberrant biliary ducts are usually found in the outer layer. However, in our patient, it was difficult to distinguish these two layers intraoperatively. Therefore, we tried to avoid an injury to the aberrant B5 by dissecting the gallbladder from the subserosal layer, staying as close to the gallbladder as we possibly could. This allowed us to perform LC safely and resulted in an excellent postoperative clinical course with a rapid recovery of the patient.

We performed LC safely in a patient with cholelithiasis and an aberrant B5 that was preoperatively accurately diagnosed with MRCP and DIC-CT, allowing us to perform the procedure safely and avoid intra- and postoperative complications.

## Data Availability

Not applicable.

## Abbreviations

B5: Biliary duct of segment 5
CT: Computed tomography
DIC-CT: Drip infusion cholecystocholangiography CT
ERCP: Endoscopic retrograde cholangiopancreatography
LC: Laparoscopic cholecystectomy
MRCP: Magnetic resonance cholangiopancreatography
